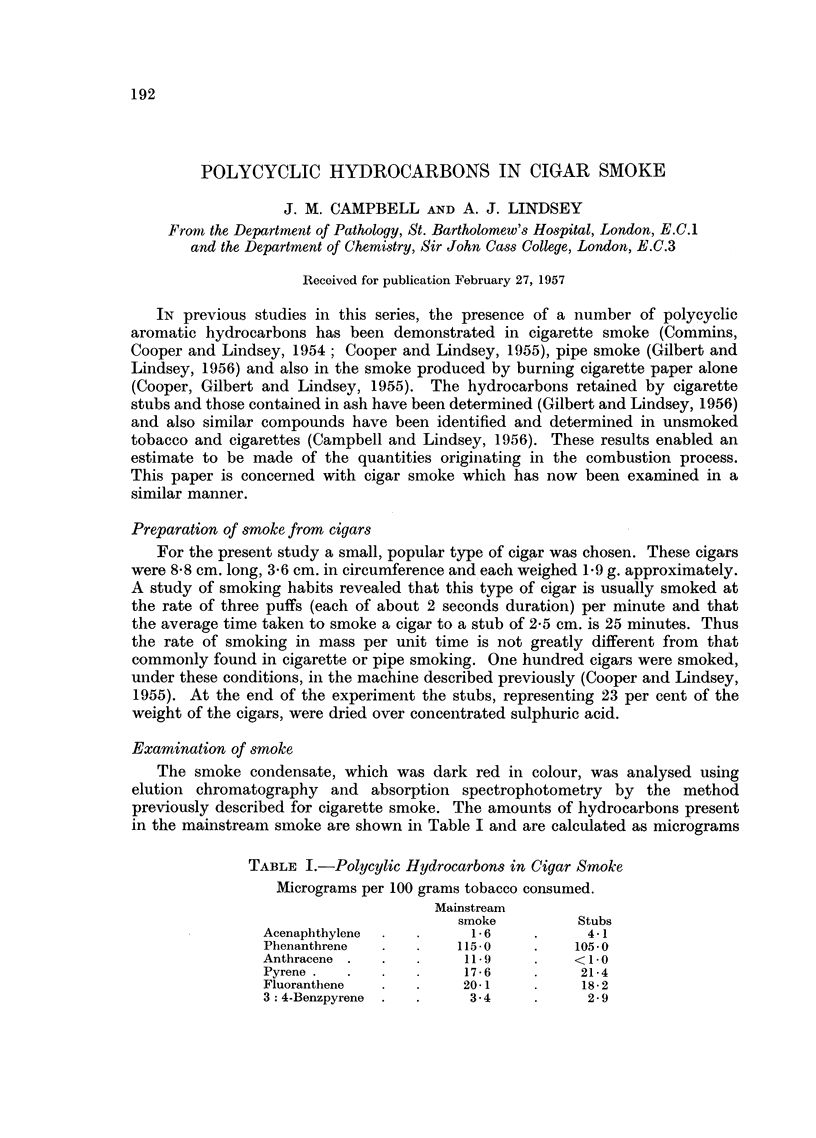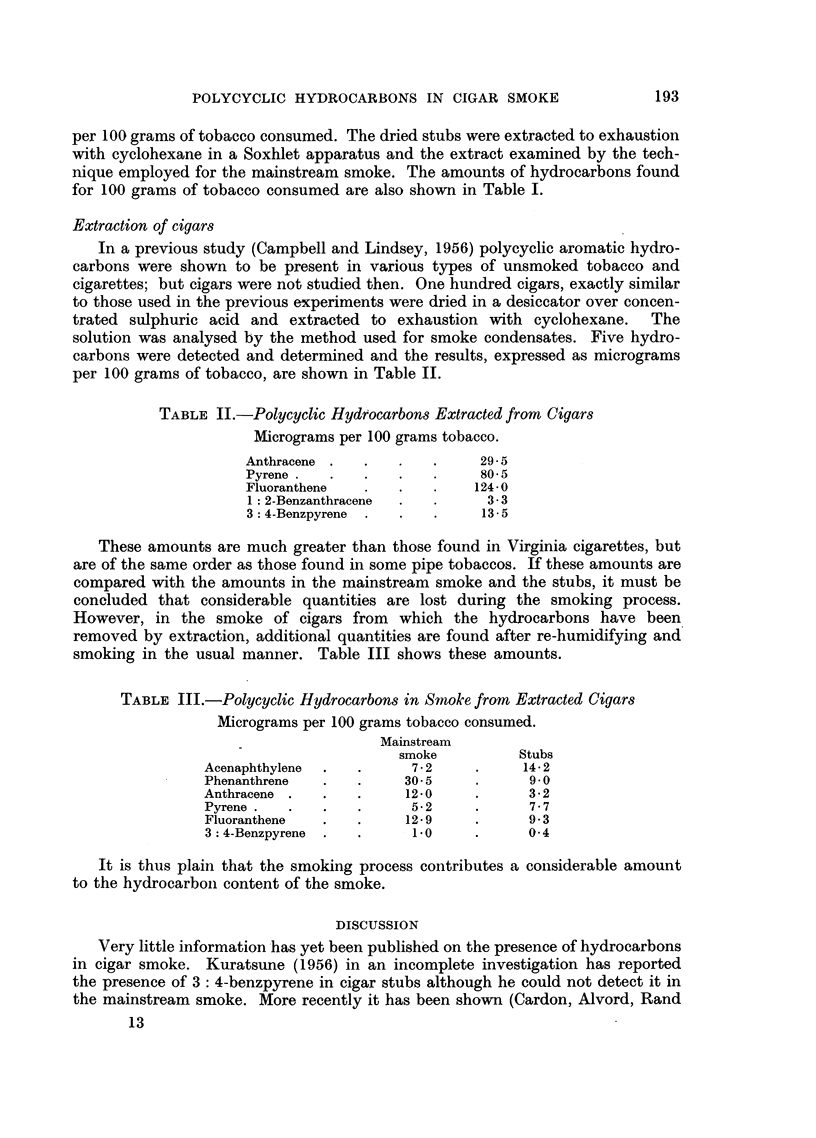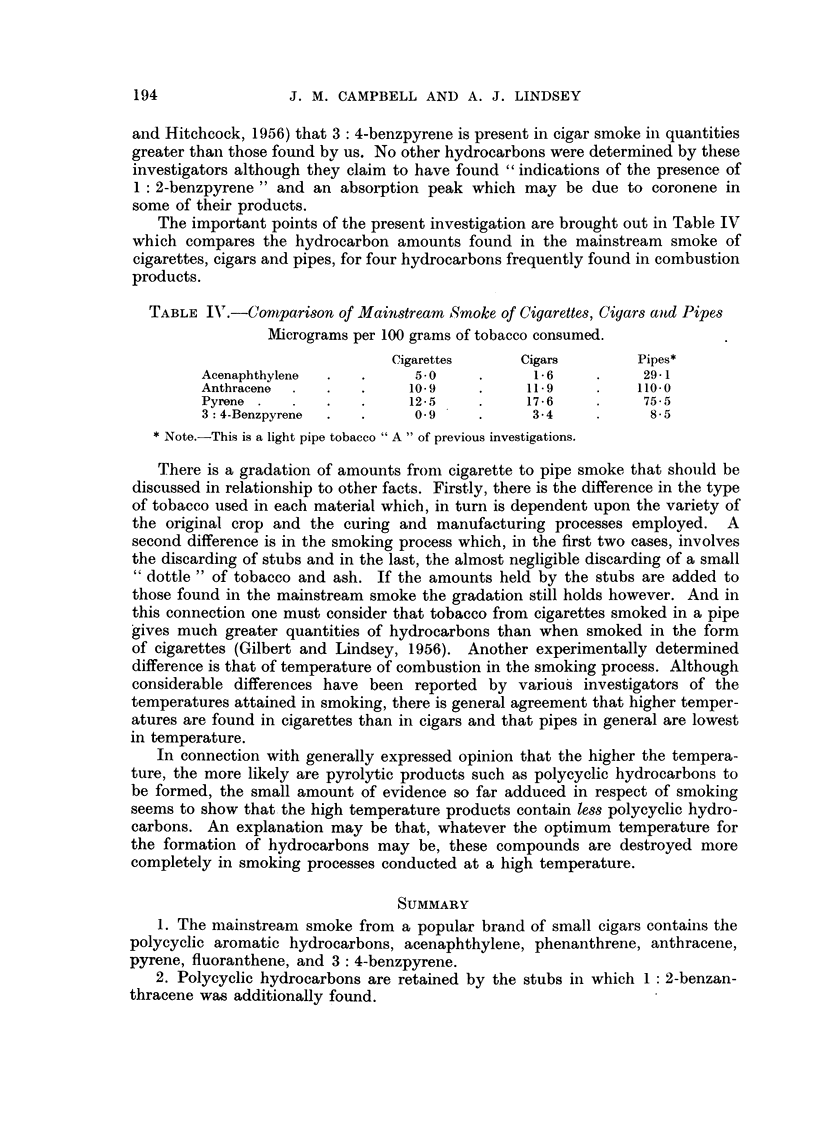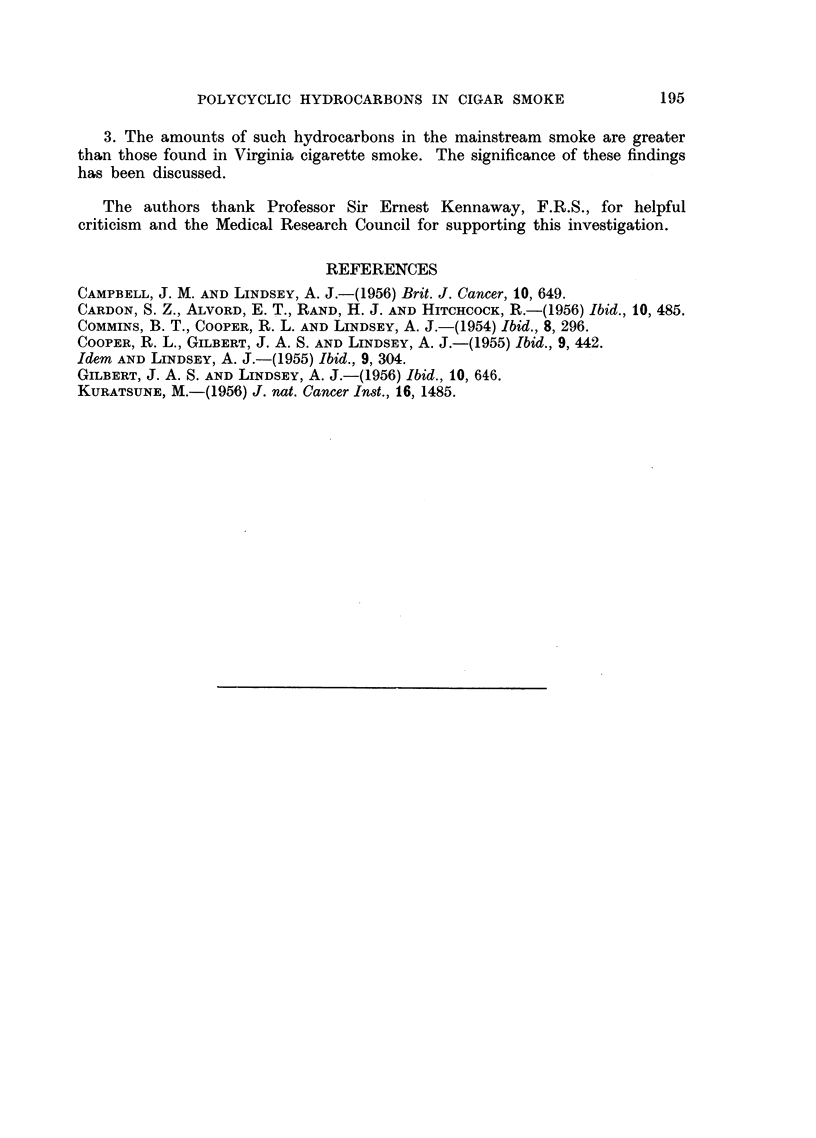# Polycyclic Hydrocarbons in Cigar Smoke

**DOI:** 10.1038/bjc.1957.26

**Published:** 1957-06

**Authors:** J. M. Campbell, A. J. Lindsey


### POLYCYCLIC HYDROCARBONS IN CIGAR SMOKE

J. M. CAMPBELL AND A. J. LINDSEY

*From the Department of Pathology, St. Bartholomew's Hospital, London, E.C.1 and the Department of Chemistry, Sir John Cass College, London, E.C.3*

Received for publication February 27, 1957

IN previous studies in this series, the presence of a number of polycyclic aromatic hydrocarbons has been demonstrated in cigarette smoke (Commins, Cooper and Lindsey, 1954 ; Cooper and Lindsey, 1955), pipe smoke (Gilbert and Lindsey, 1956) and also in the smoke produced by burning cigarette paper alone (Cooper, Gilbert and Lindsey, 1955). The hydrocarbons retained by cigarette stubs and those contained in ash have been determined (Gilbert and Lindsey, 1956) and also similar compounds have been identified and determined in unsmoked tobacco and cigarettes (Campbell and Lindsey, 1956). These results enabled an estimate to be made of the quantities originating in the combustion process. This paper is concerned with cigar smoke which has now been examined in a similar manner.

*Preparation of smoke from cigars*

For the present study a small, popular type of cigar was chosen. These cigars were 8·8 cm. long, 3·6 cm. in circumference and each weighed 1·9 g. approximately. A study of smoking habits revealed that this type of cigar is usually smoked at the rate of three puffs (each of about 2 seconds duration) per minute and that the average time taken to smoke a cigar to a stub of 2·5 cm. is 25 minutes. Thus the rate of smoking in mass per unit time is not greatly different from that commonly found in cigarette or pipe smoking. One hundred cigars were smoked, under these conditions, in the machine described previously (Cooper and Lindsey, 1955). At the end of the experiment the stubs, representing 23 per cent of the weight of the cigars, were dried over concentrated sulphuric acid.

*Examination of smoke*

The smoke condensate, which was dark red in colour, was analysed using elution chromatography and absorption spectrophotometry by the method previously described for cigarette smoke. The amounts of hydrocarbons present in the mainstream smoke are shown in Table I and are calculated as micrograms

TABLE I.—*Polycylic Hydrocarbons in Cigar Smoke*

Micrograms per 100 grams tobacco consumed.

| | Mainstream smoke | Stubs |
|---|---|---|
| Acenaphthylene | 1·6 | 4·1 |
| Phenanthrene | 115·0 | 105·0 |
| Anthracene | 11·9 | <1·0 |
| Pyrene | 17·6 | 21·4 |
| Fluoranthene | 20·1 | 18·2 |
| 3 : 4-Benzpyrene | 3·4 | 2·9 |

per 100 grams of tobacco consumed. The dried stubs were extracted to exhaustion with cyclohexane in a Soxhlet apparatus and the extract examined by the technique employed for the mainstream smoke. The amounts of hydrocarbons found for 100 grams of tobacco consumed are also shown in Table I.

*Extraction of cigars*

In a previous study (Campbell and Lindsey, 1956) polycyclic aromatic hydrocarbons were shown to be present in various types of unsmoked tobacco and cigarettes; but cigars were not studied then. One hundred cigars, exactly similar to those used in the previous experiments were dried in a desiccator over concentrated sulphuric acid and extracted to exhaustion with cyclohexane. The solution was analysed by the method used for smoke condensates. Five hydrocarbons were detected and determined and the results, expressed as micrograms per 100 grams of tobacco, are shown in Table II.

TABLE II.—*Polycyclic Hydrocarbons Extracted from Cigars*

Micrograms per 100 grams tobacco.

| | |
|---|---|
| Anthracene | 29·5 |
| Pyrene | 80·5 |
| Fluoranthene | 124·0 |
| 1 : 2-Benzanthracene | 3·3 |
| 3 : 4-Benzpyrene | 13·5 |

These amounts are much greater than those found in Virginia cigarettes, but are of the same order as those found in some pipe tobaccos. If these amounts are compared with the amounts in the mainstream smoke and the stubs, it must be concluded that considerable quantities are lost during the smoking process. However, in the smoke of cigars from which the hydrocarbons have been removed by extraction, additional quantities are found after re-humidifying and smoking in the usual manner. Table III shows these amounts.

TABLE III.—*Polycyclic Hydrocarbons in Smoke from Extracted Cigars*

Micrograms per 100 grams tobacco consumed.

| | Mainstream smoke | Stubs |
|---|---|---|
| Acenaphthylene | 7·2 | 14·2 |
| Phenanthrene | 30·5 | 9·0 |
| Anthracene | 12·0 | 3·2 |
| Pyrene | 5·2 | 7·7 |
| Fluoranthene | 12·9 | 9·3 |
| 3 : 4-Benzpyrene | 1·0 | 0·4 |

It is thus plain that the smoking process contributes a considerable amount to the hydrocarbon content of the smoke.

### DISCUSSION

Very little information has yet been published on the presence of hydrocarbons in cigar smoke. Kuratsune (1956) in an incomplete investigation has reported the presence of 3 : 4-benzpyrene in cigar stubs although he could not detect it in the mainstream smoke. More recently it has been shown (Cardon, Alvord, Rand

13

and Hitchcock, 1956) that 3 : 4-benzpyrene is present in cigar smoke in quantities greater than those found by us. No other hydrocarbons were determined by these investigators although they claim to have found "indications of the presence of 1 : 2-benzpyrene" and an absorption peak which may be due to coronene in some of their products.

The important points of the present investigation are brought out in Table IV which compares the hydrocarbon amounts found in the mainstream smoke of cigarettes, cigars and pipes, for four hydrocarbons frequently found in combustion products.

TABLE IV.—*Comparison of Mainstream Smoke of Cigarettes, Cigars and Pipes*

Micrograms per 100 grams of tobacco consumed.

| | Cigarettes | Cigars | Pipes* |
|---|---|---|---|
| Acenaphthylene | 5·0 | 1·6 | 29·1 |
| Anthracene | 10·9 | 11·9 | 110·0 |
| Pyrene | 12·5 | 17·6 | 75·5 |
| 3 : 4-Benzpyrene | 0·9 | 3·4 | 8·5 |

* Note.—This is a light pipe tobacco "A" of previous investigations.

There is a gradation of amounts from cigarette to pipe smoke that should be discussed in relationship to other facts. Firstly, there is the difference in the type of tobacco used in each material which, in turn is dependent upon the variety of the original crop and the curing and manufacturing processes employed. A second difference is in the smoking process which, in the first two cases, involves the discarding of stubs and in the last, the almost negligible discarding of a small "dottle" of tobacco and ash. If the amounts held by the stubs are added to those found in the mainstream smoke the gradation still holds however. And in this connection one must consider that tobacco from cigarettes smoked in a pipe gives much greater quantities of hydrocarbons than when smoked in the form of cigarettes (Gilbert and Lindsey, 1956). Another experimentally determined difference is that of temperature of combustion in the smoking process. Although considerable differences have been reported by various investigators of the temperatures attained in smoking, there is general agreement that higher temperatures are found in cigarettes than in cigars and that pipes in general are lowest in temperature.

In connection with generally expressed opinion that the higher the temperature, the more likely are pyrolytic products such as polycyclic hydrocarbons to be formed, the small amount of evidence so far adduced in respect of smoking seems to show that the high temperature products contain *less* polycyclic hydrocarbons. An explanation may be that, whatever the optimum temperature for the formation of hydrocarbons may be, these compounds are destroyed more completely in smoking processes conducted at a high temperature.

### SUMMARY

1. The mainstream smoke from a popular brand of small cigars contains the polycyclic aromatic hydrocarbons, acenaphthylene, phenanthrene, anthracene, pyrene, fluoranthene, and 3 : 4-benzpyrene.

2. Polycyclic hydrocarbons are retained by the stubs in which 1 : 2-benzanthracene was additionally found.

3. The amounts of such hydrocarbons in the mainstream smoke are greater than those found in Virginia cigarette smoke. The significance of these findings has been discussed.

The authors thank Professor Sir Ernest Kennaway, F.R.S., for helpful criticism and the Medical Research Council for supporting this investigation.

### REFERENCES

CAMPBELL, J. M. AND LINDSEY, A. J.—(1956) *Brit. J. Cancer*, **10**, 649.
CARDON, S. Z., ALVORD, E. T., RAND, H. J. AND HITCHCOCK, R.—(1956) *Ibid.*, **10**, 485.
COMMINS, B. T., COOPER, R. L. AND LINDSEY, A. J.—(1954) *Ibid.*, **8**, 296.
COOPER, R. L., GILBERT, J. A. S. AND LINDSEY, A. J.—(1955) *Ibid.*, **9**, 442.
*Idem* AND LINDSEY, A. J.—(1955) *Ibid.*, **9**, 304.
GILBERT, J. A. S. AND LINDSEY, A. J.—(1956) *Ibid.*, **10**, 646.
KURATSUNE, M.—(1956) *J. nat. Cancer Inst.*, **16**, 1485.